# Effect of gastric residual volume monitoring on incidence of ventilator-associated pneumonia in mechanically ventilated patients admitted to intensive care unit

**DOI:** 10.12669/pjms.36.2.1321

**Published:** 2020

**Authors:** Elnaz Faramarzi, Ata Mahmoodpoor, Hadi Hamishehkar, Kamran Shadvar, Afshin Iranpour, Tara Sabzevari, Sarvin Sanaie

**Affiliations:** 1 Elnaz Faramarzi, Liver and Gastrointestinal Disease Research Center, Tabriz University of Medical Sciences, Tabriz, Iran; 2 Ata Mahmoodpoor, Evidence Based Medicine Research Center, Tabriz University of Medical Sciences, Tabriz, Iran; 3 Hadi Hamishehkar, Department of Clinical Pharmacy, Tabriz University of Medical Sciences, Tabriz, Iran; 4 Kamran Shadvar, Anesthesiology Department, Faculty of Medicine, Tabriz University of Medical Sciences, Tabriz, Iran; 5 Afshin Iranpour, Department of Anesthesiology, Al Garhoud Private Hospital, Dubai, UAE; 6 Tara Sabzevari, Student Research Committee, Tabriz University of Medical Sciences, Tabriz, Iran; 7 Sarvin Sanaie, Aging Research Institute, Tabriz University of Medical Sciences, Tabriz, Iran

**Keywords:** Gastric, Residual volume, Intensive Care Unit, Ventilator-associated pneumonia

## Abstract

**Objectives:**

The value of gastric residual volume (GRV) monitoring in ventilator-associated pneumonia (VAP) has frequently been questioned in the past years. In this trial, the effect of GRV on the frequency of VAP was evaluated in critically ill patients under mechanical ventilation.

**Methods:**

This descriptive study was carried out on 150 adult patients admitted to the intensive care unit over a 14-month period, from October 2015 to January 2017. GRV was measured every three hours, and gastric intolerance was defined as GRV>250 cc. The incidence of vomiting and VAP, GRV, length of mechanical ventilation and ICU stay, APACHE II and SOFA scores, and mortality rate were noted.

**Results:**

The mean APACHEII and SOFA scores, ICU length of stay, and duration of mechanical ventilation in the GRV>250ml group were significantly higher than in the GRV≤250 ml group (P<0.05). Also, a significantly higher number of patients in the GRV>250ml group experienced infection (62.3%) and vomiting (71.7%) compared with the GRV≤250 group (P<0.01). The highest OR was observed for SOFA score >15 and APACHE II >30, which increased the risk of GVR>250 ml by 10.09 (1.01-99.97) and 8.78 (1.49-51.58), respectively. Moreover, the increase in GVR was found to be higher in the non-survivor than in the survivor group.

**Conclusion:**

Increased GRV did not result in increased rates of VAP, ICU length of stay, and mortality. Therefore, the routine measurement of GRV as an important element of the VAP prevention bundle is not recommended in critically ill patients.

## INTRODUCTION

Early enteral nutrition is the standard metabolic support in critically ill patients under mechanical ventilation. In patients whose nutritional requirements cannot be met by oral feeding, enteral feeding (EN) is the preferred route of nutrition support.^1^ On the other hand, more than 50% of patients in ICU have gastric dysmotility, which leads to slow gastric emptying.^2^ Delayed gastric emptying can induce several problems, which can influence ICU outcomes and lead to inadequate caloric intake or infrequent usage of enteral nutrition. Nausea, regurgitation, and aspiration can increase the risk of ventilator-associated pneumonia (VAP).^3^-^5^ Thus, monitoring of gastric residual volume (GRV) is recommended to decrease the incidence of these complications. Therefore, in cases of high GRV, decreasing the volume of enteral feeding or the formula osmolality seems to be necessary. Several studies have addressed controversial issues on the monitoring of gastric residual volume in critically ill patients receiving mechanical ventilation.^6^,^7^ Previous studies that reported a relationship between GRV and VAP were not well designed to show GRV as a reliable marker of increased risk of VAP.^8^ Recently, Kuppinger et al.^9^ reported that controlling GRV in mechanically ventilated patients is not necessary and provides no extra benefit for these patients. In the above-mentioned studies, the effects of confounding factors were not adjusted, thus the results should be warily interpreted.

According to previous findings, the use of GRV as a good indicator of the complication rate of ICU patients is debatable.^8^ Thus, the present study evaluated the relationship between GRV and ventilator-associated pneumonia in critically ill patients.

## METHODS

This prospective cross sectional study was carried out on 150 adult patients admitted to the intensive care unit over a 14-month period, from October 2015 to January 2017. This study was approved by the hospital ethics committee (Ref. 54/2648) After obtaining informed consent form patients or their next of kin 150 patients who were mechanically ventilated and were receiving enteral nutrition (EN) were enrolled in this prospective study. Inclusion criteria was the duration of mechanical ventilation of more than 48 hours, age more than 18 year old. Sample size was estimated based on the study of Tume et al.^10^ which was conducted to detect the value of caloric intake and for detection of 10% decrease in the incidence of VAP The exclusion criteria were history of esophageal gastrointestinal bleeding and surgery, intestinal obstruction, enteral feeding through a jejunostomy tube, acute pancreatitis, and pregnancy. EN was given by using a nasogastric tube. The energy requirement of the patients was calculated as 25kcal/kg/d. All patients received standard enteral formula (Enteral meal, 1kcal/1 ml, consist of carbohydrate, protein, lipid, minerals and micronutrients). All study participants received EN as an intermittent feeding, at seven feedings in 24 hours. Enteral feeding was initiated at 50ml/3h and increased by 20 ml/h every 3h until the target rate was achieved in 48-72 hours. All patients were fed in a semi-recumbent position and received mouthwashes with chlorhexidine every 8h and received pantoprazole as stress ulcer prophylaxy.

GRV was measured every three hour until the end of enteral feeding by aspiration with a 50-ml syringe. Intolerance was defined as GRV>250 cc or presence of vomiting. If the GRV was less than 250cc, the aspirated residual was regiven to the patient, and feeding was restarted. If patients had GRV of more than 250 ml we used metoclopramide and erythromycin as prokinetic drug. We initially used metoclopramide and then started with erythromycin if patients didn’t response to the metoclopramide. The next step was combination of two drugs and finally we decrease the amount of emteral nutrition to overcome the high amount of GRV. Subglottic secretion drainage was carried out through the suction port of the Taper Guard Evac endotracheal tubes in all patients. The tracheal cuff pressure was continuously monitored and maintained at the level of 20-30cm H_2_O. VAP was diagnosed based on the presence of a new or progressive pulmonary infiltrate on chest radiograph plus the existence of at least two of the following: body temperature >38.3ºC or <35.5ºC, leukocytosis (WBC>12000) or leukopenia (WBC<4000), and purulent tracheobronchial secretions. The diagnosis was confirmed by a positive culture of the tracheal aspirate >10^5^ CFU/ml or bronchoalveolar lavage (BAL) cultures growing at >10^4^ CFU/ml.

The following demographic data were collected: age, gender, primary ICU admission diagnosis, energy requirements, VAP, GRV, diarrhea, vomiting, prokinetic therapy, length of ICU and hospital stays, sequential organ failure assessment (SOFA) score, acute physiology and chronic health evaluation (APACHE) II score, length of mechanical ventilation, mortality rate, infection rate and type of organism, comorbidity, and serum levels of lactate, albumin, and CRP.

### Statistical analysis

Data were analyzed by using the Statistical Package for the Social Sciences (SPSS, version 11.5; Chicago, IL). Descriptive statistics (frequencies, percentages, and means ±SD) were reported. Chi-square test, Mann-Whitney U test, and independent *t* test were used to compare qualitative and quantitative characteristics, respectively. Logistic regression analysis was applied in estimating the crude and adjusted odds ratios (OR) and their corresponding 95% confidence intervals (95%CI). A *P-*value < 0.05 was considered as significant.

## RESULTS

The baseline characteristics of the patients are shown in Table-I.. The mean age was 57.72±19.01 years. Of the 150 patients, 95 (63.3%) were male; 54% and 49.3%, respectively, had respiratory and heart diseases at the time of admission.

**Table-I:** Baseline characteristics of patients.

Variable	Mean±SD
Age (year)	57.72±19.01
Alb(gr/dl)	3.24±0.4
BUN (mg/dl)	27.51±8.4
Cr (mg/dl)	1.46±0.52
Lactate (mmol/l)	2.43±0.55
APACHE II	25.14±5.86
SOFA	11.45±2.24
Energy intake (kcal)	1784.90±184.23

	*N(%)*

***Gender***	
Male	95(63.3)
Female	55(36.7)
***Past Medical history***	
Respiratory disease	81(54)
Heart Disease	74(49.3)
Liver disease	6(0.4)
Renal Disease	37(24.7)
Diabetes	53(35.3)
Cancer	41(27.3)
***Cause of admission***	
Poly trauma	24(16)
Cerberovascular accident	17(11.3)
Malignancy	21(14)
Sepsis/septic shock/infection	13(8.7)
Emboli syndrome	13(8.7)
Cardiorespiratory disease	37(24.7)
Other	25(16.7)

Values for age, albumin, BUN, Cr, lactate, APACHE, SOFA and energy intake was the mean value or measured variables during study period.

As indicated in Table-II, the mean APACHEII, SOFA, ICU length of stay, and duration of mechanical ventilation in the GRV>250ml group were significantly higher than in the GRV≤250 ml group (P<0.05). In addition, the findings of the Mann-Whitney U test showed that the severity of the disease based on the SOFA and APACHEII classifications was greater in patients with GRV>250 ml than in those with GRV≤250 ml (P<0.001). A significantly higher number of patients in the GRV>250ml group experienced infection (62.3%) and vomiting (71.7%) compared with the GRV≤250 group (P<0.01). The findings indicated a mortality rate of 35.8% in the GRV>250 ml group versus 14.43% in the GRV≤250 ml group. Moreover, GRV>250 ml was found to increase the mortality rate by 3.23(1.47-7.26). However, this association decreased (OR=2.29;95%CI:0.82-6.44) after adjustment for age, gender, BUN, Cr, Alb, and CRP.

**Table-II:** Comparison of diseases severity factors and ICU outcomes on the basis of gastric residual volume.

	GVR≤250 ml (n=97)	GVR>250ml (n=53)	P

	Mean±SD	Mean±SD	
APACHE II	23.52±5.08	28.15±5.85	* *<0.001
SOFA	11.03±2.07	12.23±2.36	* *0.002
ICU LoS*	10.83±4.15	12.96±5.86	* *0.01
Duration of mechanical ventilation (day)	6.78±3.17	8.71±4.33	* *0.002

	*^#^N(%)*	*^#^N(%)*	

***Classification of SOFA Score***			
6-10	38(39.2)	6(11.3)	^§^<0.001
11-15	57(58.8)	42(79.2)	
>15	2(2.1)	4(7.5)	
***Classification of APACHEII***			
15-20	26(26.8)	6(11.3)	^§^<0.001
21-30	62(63.9)	28(52.8)	
>30	9(9.3)	18(34)	
Prokinetic drugs use	55(57.3)	37(69.8)	^§§^0.16
VAP^¶^	21(21.6)	15(28.3)	^§§^0.42
Infection	33(34)	33(62.3)	^§§^0.001
Diarrhea	32(33.7)	23(44.2)	^§§^0.21
Vomiting	43(44.8)	38(71.7)	^§§^0.002
***Mortality Rate***			
Survivor	83(85.56)	34(64.2)	^§§^0.004
No Survivor	14(14.43)	19(35.8)	

* LoS: length of stay;

# Number (percent),

¶ VAP: ventilator-associated pneumonia;

* * P value: Comparison within group by independent - test;

§ P value: comparison between group by Mann-Whitney U test,

§§ P-value: Comparison between group by χ^2^ test.

The association between disease severity and GRV is presented in Table-III. In addition, incidence of infection and vomiting was significantly correlated with GRV>250 ml. However, these correlations decreased after adjusting for age, gender, BUN, Cr, CRP, Alb, and prokinetic drug use. The highest OR was observed for SOFA scores>15 and APACHE II scores>30, which increased the risk of GRV>250 ml by 10.09 (1.01-99.97) and 8.78 (1.49-51.58), respectively.

**Table-III:** Association of disease severity with gastric residual volume.

	GVR≥250ml			

	Unadjusted OR(95%CI)	P	*Adjusted OR(95%CI)	P
Infection	3.2(1.95-6.42)	0.001	2.04(0.77-5.39)	0.15
Vomiting	3.12(1.51-6.41)	0.002	1.63(0.63-4.23)	0.30
Diarrhea	1.56(0.78-3.12)	0.20	1.10(0.44-2.73)	0.82
VAP	1.41(0.65-3.04)	0.38	0.78(0.27-2.18)	0.63
Mortality	3.27(1.47-7.26)	0.004	1.96(0.73-5.28)	0.18
***Classification of SOFA Score***				
6-10	Reference			
11-15	4.66(1.8-12.05)	0.001	5.36(1.40-20.50)	0.01
>15	12.66(1.88-84.96)	0.009	10.09(1.01-99.97)	0.04
***Classification of APACHEII***				
15-20	Reference			
21-30	1.95(0.72-5.28)	0.18	3.51(0.73-16.96)	0.01
>30	8.66(2.62-28.63)	<0.001	8.78(1.49-51.58)	0.004

* Adjusted for age, gender, BUN, Cr, CRP, Albumin, Prokinetic drugs use.

**Fig 1:**
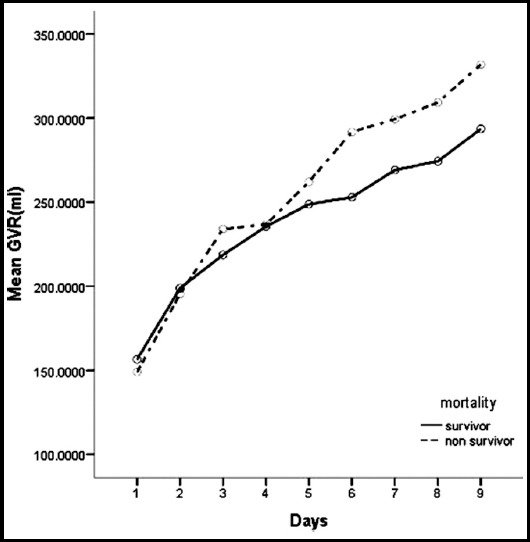
Comparison of gastric residual volume changes during the study between survivors and non-survivors

The comparison of gastric residual volume changes during the study between survivors and non-survivors is shown in Fig.-1. The data indicate similar GRV between survivors and non-survivors. The mean GRV increased significantly (P<0.001) in both groups during the study. However, the increase in GRV was found to be higher in the non-survivor group than in the survivor group.

## DISCUSSION

The results showed that, after adjustment for confounding factors, GRV of more than 250 ml did not have a significant effect on VAP and mortality in critically ill patients. The most important factor for GRV is the severity score of patients: the higher score and the greater the GRV. The value of periodic GRV measurements in decreasing the risk of VAP incidence has frequently been questioned in the past years. Previous studies recommended lower volume of GRV for intolerance in surgical patients and showed higher percentage of VAP with GRV in surgical patients which is opposite to our results, as most of our patients were surgical patients. Increasing the GRV threshold before interrupting gastric feeding results in marginal increases in EN delivery. A recently published meta-analysis from six RCT and six observational studies showed that the use of a lower gastric residual volume cutoff is not recommended.^10^ In patients who receive mechanical ventilation, routine monitoring of GRV is not recommended and leads to a decreased nursing workload. Nevertheless, the increased amount of delivered calories could not be proven to lead to improved survival.^11^ Of the six observational studies, only one adjusted the outcome based on confounding risk factors, thus the difficulty in interpreting the results.^3^ In the above-mentioned study, the authors showed that the frequency of aspiration significantly increased with GRV of more than 250 ml or GRV of more than 200 ml if repeated.^3^ Among the RCTs, only two were of high quality. These showed that increased GRV did not lead to adverse complications. However, the nurses were not blinded to the group assignments; hence, the patients in the intervention group received only about 200 kcal more during the first week after randomization.^11^,^12^ In their review, Metheny et al. showed that GRV of less than 200 ml seemed to be well tolerated and that values within the range of 200-500 ml should be considered as a potential risk factor for VAP; however, in cases of GRV >500 ml, feeding should be stopped, especially during regurgitation or aspiration.^12^ The results of a review showed that EN should be stopped only during overt aspiration and regurgitation; thus, routine monitoring of GRV was not recommended,^13^ which did not contribute to inadequate feeding. Ozen et al. showed that the discrepancies in the measurement of GRV make such practice unreliable in monitoring feeding intolerance and that the use of GRV can be discontinued as a standard preventive strategy in medical ICUs; however, surgical patients may benefit from a lower GRV threshold.^14^ Juve-Udina et al. recommended the reintroduction of gastric aspirate at up to 250 ml per check in critically ill patients to achieve a more physiologic gastric content management approach without any significant change in the risk of any severe adverse complications while considering dysglycemia.^15^

Several factors may explain the results of the present research regarding GRV, which are consistent with previous studies. First, gastric residual volume does not have a standardized definition, and the method of its assessment based on aspiration is dependent on the tube size and position and the nurses’ experience.^16^ Second, the optimal GRV cutoff that leads to vomiting/regurgitation is not defined. Here, 250 ml was used as the cutoff value for intolerance based on the literature.^17^ However, previous results have indicated that amounts of less than 250 ml were not associated with decreased VAP rates,^18^,^19^ and other studies have shown that GRV >500 ml was not associated with increased VAP rate.^20^ Third, in the pathogenesis of VAP, oropharyngeal secretions and their leakage around the ETT cuff is the main mechanism for VAP; however, for the gastropulmonary route, there are several controversial results, such as in trials on sucralfate^21^,^22^ and continuous enteral feeding versus intermittent enteral feeding.^19^ The present results showed that higher APACHE II and SOFA scores are associated with higher GRV, which remained significant after adjustment, especially with APACHE II scores>30 and SOFA scores>15.

In addition, enteral nutrition, which is lost by vomiting or being discarded, was not measured and led to overestimation of delivered calories and increased morbidity and mortality. The results showed that increased GRV is not associated with significant mortality because the VAP pathogenesis involves many factors of which GRV is only one. On the other hand, the compliance of healthcare workers with VAP bundle criteria is very important regarding VAP frequency. In our ICU, the compliance with VAP bundle criteria is high at almost 85%. Because GRV monitoring is a time-consuming process, its removal from the VAP bundle would allow an increased focus on interventions proven to decrease the risk of VAP.

### Limitations of the study

The present study was carried out in two ICUs (university-affiliated) with surgical patients with a limited sample size; thus, our results cannot be generalized to all critically ill patients. Also, 250 ml was used as the cutoff for gastric intolerance and high GRV. More studies having larger sample sizes and applying other cutoff values for high gastric residual volume are therefore necessary. Another limitation of this study was that blinding of ICU staff to the group assignments was not possible. However, previous reports have shown that an unblinded design has little or no effect on vomiting rates.^10^

## CONCLUSION

Frequent assessment of GI tolerance to tube feedings is a crucial element of practice. The results of the present study showed that increased GRV did not result in increased rates of VAP, ICU length of stay, and mortality. Therefore, the routine measurement of GRV as an important element of the VAP prevention bundle is not recommended in critically ill patients, and its removal from the bundle allows an increased focus on interventions proven to decrease the risk of VAP. This can lead to better optimization of enteral nutrition to meet the caloric targets and avoid underfeeding in these patients.
